# Modulating Central Gain in Tinnitus: Changes in Nitric Oxide Synthase in the Ventral Cochlear Nucleus

**DOI:** 10.3389/fneur.2015.00053

**Published:** 2015-03-10

**Authors:** Ben Coomber, Victoria L. Kowalkowski, Joel I. Berger, Alan Richard Palmer, Mark Nelson Wallace

**Affiliations:** ^1^MRC Institute of Hearing Research, Nottingham, UK; ^2^Otology and Hearing, Division of Clinical Neuroscience, University of Nottingham, Nottingham, UK

**Keywords:** nitric oxide synthase, tinnitus, acoustic over-exposure, cochlear nucleus, guinea pig

## Abstract

A significant challenge in tinnitus research lies in explaining how acoustic insult leads to tinnitus in some individuals, but not others. One possibility is genetic variability in the expression and function of neuromodulators – components of neural signaling that alter the balance of excitation and inhibition in neural circuits. An example is nitric oxide (NO) – a free radical and potent neuromodulator in the mammalian brain – that regulates plasticity via both pre-synaptic and postsynaptic mechanisms. Changes in NO have previously been implicated in tinnitus generation, specifically in the ventral cochlear nucleus (VCN). Here, we examined nitric oxide synthase (NOS) – the enzyme responsible for NO production – in the guinea pig VCN following acoustic trauma. NOS was present in most cell types – including spherical and globular bushy cells, small, medium, and large multipolar cells, and octopus cells – spanning the entire extent of the VCN. The staining pattern was symmetrical in control animals. Unilateral acoustic over-exposure (AOE) resulted in marked asymmetries between ipsilateral and contralateral sides of the VCN in terms of the distribution of NOS across the cochlear nuclei in animals with behavioral evidence of tinnitus: fewer NOS-positive cells and a reduced level of NOS staining was present across the whole extent of the contralateral VCN, relative to the ipsilateral VCN. The asymmetric pattern of NOS-containing cells was observed as early as 1 day after AOE and was also present in some animals at 3, 7, and 21 days after AOE. However, it was not until 8 weeks after AOE, when tinnitus had developed, that asymmetries were significant overall, compared with control animals. Asymmetrical NOS expression was not correlated with shifts in the threshold hearing levels. Variability in NOS expression between animals may represent one underlying difference that can be linked to whether or not tinnitus develops after noise exposure.

## Introduction

The neural basis of tinnitus remains unknown. One putative model proposes increased gain in auditory brainstem neurons to compensate for reduced afferent input following peripheral deafferentation, such as occurs after an acoustic insult (1, 2). A side effect of this process is an elevation of spontaneous activity in auditory neurons. This may be perceived as a low-level sound and hence manifest as tinnitus. The idea of enhanced “central gain” is supported by evidence from animal studies. Changes in neuronal excitability in the auditory system, including core and belt regions of auditory cortex (3, 4), the inferior colliculus (5), and both the dorsal cochlear nucleus (DCN) and ventral cochlear nucleus (VCN) (6–8) have been implicated in tinnitus generation. This may occur as a result of alterations in both inhibitory and excitatory neurotransmission (9–14).

However, this model is insufficient in that it cannot explain the variable occurrence of tinnitus even when considering a single well-described etiology, e.g., why some individuals experiencing noise-induced hearing loss develop tinnitus, while others do not (15, 16). At present, the mechanisms that underlie this variability remain unclear. Neuromodulators offer one potential mechanism as they are involved in subtle interactions with groups of neurons and of modulating the balance of excitatory and inhibitory neurotransmission. One such neuromodulator is nitric oxide (NO), a ubiquitous, gaseous, signaling molecule that can act through a variety of protein targets. NO modulates synaptic plasticity to regulate neuronal activity, either increasing or decreasing excitability. It is capable of altering normal physiological signaling in the brain, such as long-term potentiation via retrograde augmentation of pre-synaptic glutamate release (17). However, uncontrolled NO release – particularly from glial cells – is often associated with inflammation and pathology [(18, 19) for a review].

Nitric oxide is produced by neuronal nitric oxide synthase (nNOS) in the brain, which is expressed in specific subsets of neurons, via a calcium-dependent signaling process. Neuronal NO production, and consequently nNOS distribution, is widespread throughout the brain, including the auditory system (20, 21). We have demonstrated previously that levels of nitric oxide synthase (NOS) are asymmetrically altered in the VCN of guinea pigs (GPs) subjected to unilateral acoustic over-exposure (AOE), and that this correlated with behavioral evidence of tinnitus (22). In addition, changes in nNOS expression have also been demonstrated in the VCN following salicylate administration in rats (23), and after removal of the cochlea in mice (24), experimental conditions also linked with tinnitus (25, 26). Thus, transformations in NO signaling could contribute to the plastic changes found in the VCN following AOE. However, to date, neither the function of NO in the CN, nor the involvement of nNOS in tinnitus generation has been studied in detail.

In the present study, we examined NOS in the VCN with the aim of identifying the types of neurons that express it, establishing the timescale over which NOS levels change after AOE, and determining how variable these changes are between animals. We also wanted to determine if the changes in NOS levels are associated with a particular sub-group of neurons. In the VCN, spherical bushy cells (SC) are predominantly associated with the anterior subdivision [anterior ventral cochlear nucleus (AVCN)], while globular bushy cells (GC) are predominantly located in the posterior subdivision [posterior ventral cochlear nucleus (PVCN) (27)]. The multipolar cells form different functional sub-classes with different projection targets and are more evenly distributed across the VCN (28, 29). Large multipolar cells (LMs) are glycinergic and provide inhibition (30), while small-to-medium-sized multipolar cells are excitatory and project mainly to the inferior colliculus, via the trapezoid body (31–33). By examining the spatial location and size distribution of NOS-containing neurons, we sought to determine if either size or location could reveal changes in a specific functional class of VCN neuron, following AOE.

## Materials and Methods

### Animals and preparation

All procedures were carried out in accordance with the European Communities Council Directive of 24 November 1986 (86/609/EEC) and the approval of the Animal Welfare and Ethical Review Body at the University of Nottingham, UK. Experiments were conducted on a total of 29 male and female pigmented GPs weighing 400–700 g at the time of AOE. A total of 24 animals were noise-exposed, while a further 5 animals were used as controls. GPs were bred in-house and group-housed on a 12:12 h light:dark cycle. Food and water were freely available.

Guinea pigs were anesthetized with Ketamine (Ketaset; 50 mg kg^−1^, i.p.; Fort Dodge Animal Health Ltd., Southampton, UK) and Xylazine (Rompun; 10 mg kg^−1^, i.p.; Bayer PLC, Newbury, UK), supplemented with further administrations of a mixture of Ketamine and Xylazine in a ratio of 15:2 (i.m.) throughout the procedure. Core body temperature was monitored throughout and maintained to 38 ± 0.5°C using a homeothermic heating pad (Harvard Apparatus Ltd., Edenbridge, UK) and a rectal probe.

### Auditory brainstem responses

Auditory brainstem responses (ABRs) were recorded to determine hearing thresholds prior to and immediately after noise exposure. An additional series of ABR recordings were performed immediately before animals were euthanized to determine hearing threshold recovery. Once anesthetized, hypodermic needles were inserted through the skin to act as recording electrodes over the right and left mastoids, and a reference needle was inserted at the vertex point. These ABR recording electrodes were connected via a Tucker Davis Technologies (TDT; Alachua, FL, USA) low-impedance headstage amplifier (RA4LI) and a TDT Medusa digitizing preamplifier (RA16PA) to a TDT System 3 interface (RX7). Auditory stimuli for ABRs were presented binaurally via 25 mm loudspeakers (Peerless DX25). To maintain a closed speaker system, polyethylene tubes (diameter of 20 mm) were connected to the speakers and positioned over the ear and pinna forming a seal around each ear. The system was calibrated using a 40BP 1/4″ pressure condenser microphone, 26AC preamplifier, and 12AR power supply (all G.R.A.S, Holte, Denmark) attached to a calibrated 1 mm diameter probe positioned near the entrance to the ear canal. GPs were placed inside a sound-proof booth and remained there for the duration of the ABR recording and AOE.

Auditory brainstem responses were recorded independently for left and right ears in response to ipsilaterally presented pure tone bursts of 5, 10, and 15 kHz (5 ms duration; rise/fall time of 1 ms), using custom-written software. Tones were presented at progressively decreasing sound levels (5–10 dB steps; starting from 90 dB SPL) until an auditory-evoked response threshold was determined based on the absence of a discernible ABR signal (1500 repeats; gain ×25,000; sampling duration 20 ms; filtered at 300 Hz–1 kHz).

### Acoustic over-exposure

After pre-trauma thresholds had been determined, the right speaker was electrically disconnected, but left attached to the tube, and the polyethylene tube was plugged with cotton wool. The right pinna was then folded over, and the plugged tube re-positioned over the ear. This served to reduce the risk of incurring hearing deficits in the right “control” ear. The left ear was then exposed to narrow band-passed noise bursts (duration of 500 ms and gaps of 200 ms; center frequency 10 kHz; bandwidth 1 kHz), presented at 120 dB SPL, for 1 h.

### Histology

Acoustic trauma was induced unilaterally, providing a within-animal control, i.e., comparing the exposed ipsilateral CN (CN_ipsi_) with the un-exposed contralateral CN (CN_contra_). The CN of noise-exposed GPs (*n* = 24) was examined for changes in NOS levels and compared with a group of un-exposed control GPs (*n* = 5). NOS levels were quantified by staining with reduced nicotinamide adenine dinucleotide phosphate diaphorase (NADPH-d), which represents the presence of all isoforms of NOS in aldehyde-fixed tissue (34, 35). To confirm that changes in NOS expression could be attributed to the neuronal form (nNOS), and were not due to alterations in inducible NOS (iNOS), endothelial NOS (eNOS), or an additional sub-type found in mitochondria [see Ref. (20)], we also performed immunohistochemical staining with an antibody specific to nNOS. This was particularly important, given that the different isoforms are not expressed exclusively in specific tissues.

Separate groups of animals were allowed to recover after AOE for 24 h (*n* = 5), 72 h (*n* = 5), 7 days (*n* = 3), or 21 days (*n* = 3). An additional group of animals was examined behaviorally for tinnitus using the gap induced prepulse inhibition of acoustic startle test, before AOE and then 8 weeks after noise exposure [behavioral data presented in Ref. (22)]. GPs were then anesthetized and ABRs measured (as described in the previous section) before an overdose of pentobarbital was administered (i.p.) and animals were transcardially perfused with 4% paraformaldehyde for histological assessment with NADPH-d [see Ref. (18)] and nNOS [see Ref. (36)]. Brains were removed from the skull, post-fixed for 12 h, and cryo-protected for 24 h in 30% sucrose dissolved in 0.1 M phosphate buffer (PB; pH 7.4). Brainstem blocks were embedded in gelatin/albumin and sectioned coronally at either 50 or 100 μm on a vibratome. Sections were initially cut at 100 μm if they were just being stained for diaphorase as this meant that all the sections from the block could easily be stained. However, the higher background and increased incidence of stained somata overlying each other meant that all the later blocks were cut at 50 μm as this made the counting easier and allowed us to stain an alternate series immunohistochemically.

Sections to be stained for NADPH-d were incubated at 37°C for 30 min in 10 ml of PB containing 7 mg nitroblue tetrazolium, 12 mg NADPH, and 0.2% Triton X-100. Sections were then washed in PB, mounted on gelatin-coated slides, air-dried for ~1 h, dehydrated in a graded series of ethanol solutions, and cover-slipped.

In four cases, alternate sections were stained for NADPH-d (as outlined above), or immuno-stained for nNOS. Sections were immersed in PB containing 0.3% H_2_O_2_ and 10% methanol, followed by PB containing 5% normal horse serum (NHS) and 0.5% Triton X-100. Sections were then incubated with monoclonal anti-nNOS primary antibody (1:2000; N2280, Sigma-Aldrich, UK) in PB containing 5% NHS and 0.5% Triton X-100 overnight at 4°C. Subsequently, sections were washed (3 × 10 min in PB), before incubation with biotinylated anti-mouse secondary antibody (1:100; Vector Laboratories, UK) for 2 h at room temperature. Sections were then washed (3 × 10 min in PB), and incubated in PB-containing Vectastain ABC *Elite* solution (Vector Laboratories) and 1% NHS. After a further series of washes (3 × 10 min in PB), the sections were placed in 0.05% diaminobenzidine (DAB) solution, in PB, for 10 min, followed by a solution of hydrogen peroxide in DAB solution for an additional 10 min. The sections were then washed in PB (3 × 10 min) to halt the DAB reaction, mounted on gelatin-coated slides, air-dried, dehydrated, and cover-slipped.

### Data analysis

In our pilot experiments, we attempted to quantify changes in the numbers of NOS-containing somata in both the VCN and DCN. However, it was difficult to identify individual neuronal somata in the dorsal subdivision due to large amounts of diaphorase in the neuropil, particularly in the granule and molecular cell layers (22). Consequently, in the present study, we focused our analyses on the anterior (AVCN) and posterior (PVCN) subdivisions of the VCN where we could clearly identify individual cells. Quantification of changes was made by measuring three variables: the numbers of NADPH-d stained cells on each side of the VCN, the cross-sectional area of the stained cells, and the luminance values of a sample of well-defined cells.

Cell counts were performed on a series of alternate VCN sections (50 μm) or every section (100 μm) by a researcher blinded to treatment group (control or AOE) using systematic random sampling. The series was started randomly near the caudal pole of PVCN and all the stained cells counted that were in a single focal plane (using a 10× Plan Apo objective) close to the center of the section. The depth of the focal plane was measured and found to be between 10 and 12 μm thick, while the sections were 20–25 μm thick after shrinkage associated with dehydration. Counts were made on optical planes that were centered 100 μm apart (before shrinkage) and involved over 30 sections per brain. Thus, we counted about one quarter of the NADPH-d-positive cells within VCN. We have not presented cell counts as absolute numbers, instead using relative values as counts varied greatly between brains, at least partly as a result of differences in the hardness of fixation. Data were normalized by presenting them as percentages of the total number of cells counted or as a ratio of the two sides. The ratio of the total number of NADPH-d-positive cells counted in the VCN_ipsi_ compared with the number counted in the VCN_contra_ was calculated for each GP, and a mean ratio value calculated by pooling across all animals within a group. These data, and all other data presented, were assessed for normality where possible with a D’Agostino-Pearson test, or otherwise with a Kolmogorov–Smirnov test. In instances where neither test could determine normality, we selected a non-parametric test as a conservative approach to avoid falsely detecting significant differences. The ipsilateral–contralateral ratio for control GPs, tinnitus GPs, and the other AOE groups was assessed statistically with a Kruskal–Wallis test and Dunn’s Multiple Comparison *post hoc* test (*P* < 0.05 for all tests). AOE groups terminated at 1, 3, 7, or 21 days after AOE were also compared for differences in hearing threshold shifts (as determined by ABR recordings) at each frequency (5, 10, or 15 kHz) with a Kruskal–Wallis test and Dunn’s Multiple Comparison *post hoc* test. The influence of hearing loss on the magnitude of ipsilateral–contralateral NADPH-d asymmetries in these groups was explored with a linear regression analysis.

Brains from a group of animals that exhibited tinnitus behavior [*n* = 8; data presented in Ref. (22)] were further compared with un-exposed control brains, and AOE-treated GPs killed at shorter time-points (1 day through to 21 days). The spatial distribution of NADPH-d-positive cells was plotted throughout posterior and anterior subdivisions of the VCN for each animal. NADPH-d distribution according to rostral–caudal position was statistically assessed for pooled control and pooled tinnitus animals separately with a two-way ANOVA and Sidak’s *post hoc* test. Based on these spatial distribution plots, we sub-divided the VCN into AVCN and PVCN sub-populations, and compared VCN_ipsi_:VCN_contra_ ratios between controls and tinnitus animals for each, using Mann–Whitney tests. To determine if changes in NADPH-d levels were associated with a particular sub-group of neurons, we measured soma area in NADPH-d-positive cells. Image processing software (Neurolucida, Microbrightfield, Colchester, VT, USA) was used to calculate the cross-sectional area of cells chosen by systematic random sampling. A grid of 5 × 2 squares was placed over the VCN, and all cells measured within a randomly chosen square. The squares were numbered 1–10 and a random number generator used to choose a square. Every second section was counted starting at the caudal pole of the VCN.

The amount of NADPH-d present in positively labeled VCN neurons was examined indirectly with microdensitometric measurements of luminance in digital images. An image from the focal plane close to the center of the section was captured and measurements were only made on cells that were in focus. As most of the cells had cell diameters of <30 μm and the sections were 50 μm thick, this ensured that the entire soma was within the section. Neurolucida software was used to provide a gray-scale value for each pixel in captured images acquired with a 10× objective lens. Luminance showed a large range of values over the soma with lower values over the nucleus and darker clumps in the cytoplasm. The value for each cell was taken as the mean value of the 20 darkest pixels that formed a contiguous group to give a representative value for the amount of enzyme in the cytoplasm. This gave a single number for each cell. Luminance values were calculated in this way for the 30 most darkly stained cells from four pairs of sections from corresponding points of the VCN on either side (120 cells in total for each side). Two of these sections were in PVCN and the other two in AVCN or the transition area above the nerve root. In order to determine whether enzyme levels in the VCN had increased or decreased after AOE, they were measured in relation to an internal standard that should not have been affected by noise exposure. Luminance measures were made on the large reticular neurons (RNs) in two bands on either side of the midline in the medulla. These bands were 0.8 mm wide and measurements were taken from 50 cells on each side using two sections at the level of the caudal pole of the DCN. A measure of reaction product density (proportional to the amount of enzyme) was obtained by taking the log_10_ of the inverse of the luminance values.

The change in the ratio of the mean densities derived from the 120 darkest cells from each side was calculated to compare VCN_ipsi_ with VCN_contra_ for each GP, and then pooled across respective groups to calculate a mean density ratio for controls and tinnitus animals. Mean densities were compared statistically with an unpaired *t*-test. For the purpose of normalizing changes in the VCN to NADPH-d density in RNs, the following statistical tests were applied: Left- and right-side RN luminance values were compared individually for each animal in the control group with a Mann–Whitney test. Median values from left- and right-side RN populations were then compared across all control animals for differences with a paired *t*-test. Staining density ratios were then calculated relative to RN for VCN_ipsi_ and VCN_contra_ in each control animal and each tinnitus animal. NADPH-d density ratios in the VCN (normalized relative to RN) in control and tinnitus animals were statistically examined with a repeated measures two-way ANOVA. The relationship between asymmetrical changes in the number of NADPH-d-stained neurons and the density of NADPH-d staining was examined across all experimental animals, i.e., controls, tinnitus animals, and the other noise-exposed groups (1, 3, 7, or 21 days), with a linear regression analysis.

## Results

### Ubiquitous NOS expression in VCN principal cells

**Figure 1:**
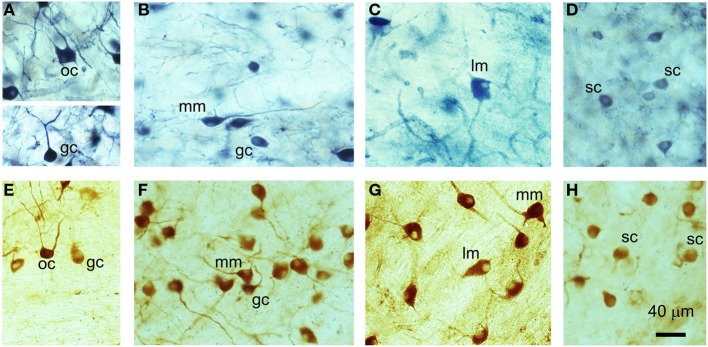
**Examples of different cell types in the VCN that have been stained for NADPH-diaphorase (A–D) or nNOS immunoreactivity (E–H)**. **(A)** Globular bushy cell (GC) and octopus cell (OC), **(B)** medium multipolar (MM) and globular bushy cell (GC), **(C)** large multipolar or giant cells (LM), **(D)** spherical bushy cell (SC), **(E)** octopus cell (OC) and globular bushy cell (GC), **(F)** medium multipolar (MM) and globular bushy cells (GC), **(G)** large multipolar (LM) and medium multipolar cells (MM), **(H)** spherical bushy cells (SC).

Nitric oxide synthase staining, whether obtained with diaphorase or a specific primary antibody, was widespread throughout the VCN. NOS was present in a variety of neuron types that included small, medium, and large cells, some of which possessed multiple dendrites, while some appeared to have only one dendrite (Figure 1). Previous work in GPs by Hackney et al. (27) indicated that rounded cells with a single dendrite in AVCN predominantly correspond to SCs, while those in the PVCN were more likely to be GCs. Here, we observed two sub-types of bushy cell in PVCN: the first with a primary dendrite that appeared to be straight and unbranched for at least 40 μm (Figure 1A), and a second sub-type with a primary dendrite that branched immediately after leaving the soma (Figure 1F) in a manner similar to SCs (Figure 1H). A large number of stained cells in the PVCN appeared to be multipolar and varied considerably in size (Figures 1B,C,F,G). In the dorsoposterior aspect of the VCN, there were occasional cells that exhibited an octopus cell morphology (Figures 1A,E). Thus, we observed examples of NOS staining in all the major cell classes in the VCN. However, NOS-stained cells only made up a proportion of all cells within any given class; many neurons were either not stained or were unidentifiable (morphologically) owing to insufficient staining in the proximal dendrites. This does not mean that the unstained cells did not contain any NOS – only that our methods were not sufficiently sensitive to detect any.

**Figure 2:**
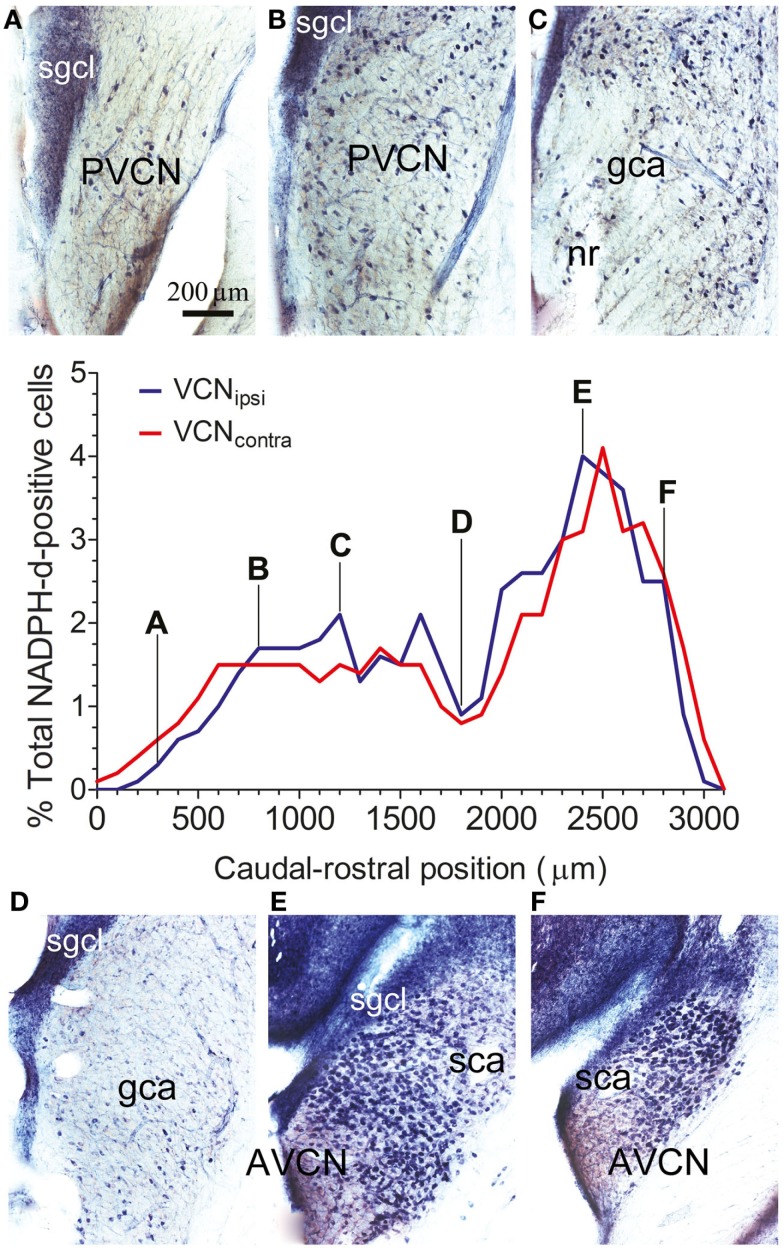
**The spatial distribution of NOS in the VCN**. The percentage of NADPH-d-positive neurons from the total population of counted NADPH-d-positive cells is shown according to position on the caudal–rostral axis of the VCN in a single control GP. Data are shown for VCN_ipsi_ (blue) and VCN_contra_ (red). Photographs show staining patterns in coronal sections at progressive points along the caudal–rostral axis of the VCN, from PVCN through to AVCN at **(A)** 300 μm, **(B)** 800 μm, **(C)** 1200 μm, **(D)** 1800 μm, **(E)** 2400 μm, and **(F)** 2800 μm, relative to the caudal pole of PVCN. Also indicated on the images are the locations of the small granule cell layer (SGCL), globular (bushy) cell area (GCA), and spherical (bushy) cell area (SCA), as per the scheme of Hackney et al. (27).

Throughout the VCN, stained somata were relatively prominent because of weak neuropil staining, with the exception of the small granule cell layer at the dorsal edge (SGCL in Figure 2). The numbers of stained cells varied depending on the rostro-caudal position of the section. For each animal, the total number of NADPH-d-stained cells was counted within a standard optical plane for each section. Cell counts for VCN_ipsi_ and VCN_contra_ were then expressed as a percentage of the total number of NADPH-d-stained cells counted, and plotted according to distance from the caudal pole (shown for a single control animal in Figure 2). A consistent bimodal distribution of stained cells was apparent, although the absolute numbers and distribution of stained cells varied between individual control animals, partly as a result of different degrees of fixation. This variation in cell numbers is illustrated in Figure 2; the numbers of stained cells was particularly high in the spherical bushy cell area (SCA) at the rostral pole in this GP where the ratio of AVCN/PVCN was 1.22. Nonetheless – and in spite of variability between animals – the distribution and numbers of stained cells were similar when comparing VCN_ipsi_ and VCN_contra_ across all control animals (see next section). The mean number of stained neurons counted in VCN_ipsi_ was 2953, while in VCN_contra_, it was 2874. This represented a difference of 3%, and the difference between the two sides in individual animals ranged from 5 to −5%.

### Altered distribution of NOS in tinnitus brains

**Figure 3:**
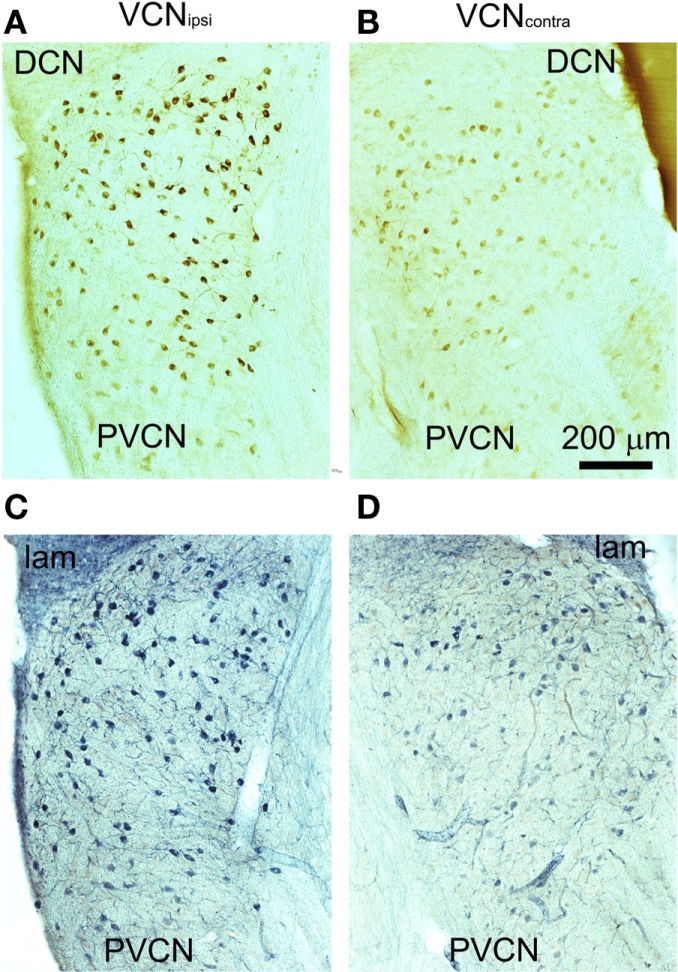
**Representative coronal sections through PVCN from an animal with behavioral evidence of tinnitus**. Sections were stained immunohistochemically for nNOS **(A,B)** or for NADPH-diaphorase **(C,D)**. Unilateral AOE leads to more densely stained neurons in the VCN_ipsi_ **(A,C)** compared with the VCN_contra_ **(B,D)**.

Behavioral testing indicated that tinnitus had developed in some animals by 8 weeks after unilateral AOE (22). In these animals, there were significant differences between VCN_ipsi_ and VCN_contra_ whereby NADPH-d-containing neuronal somata were more numerous and more densely stained in VCN_ipsi_ than in VCN_contra_ (Figure 3). The staining profiles of NADPH-d and nNOS were similar, indicating that changes in NADPH-d expression in tinnitus animals can be attributed to the neuronal isoform of the enzyme. The changes in number, staining intensity, and size will be described separately.

**Figure 4:**
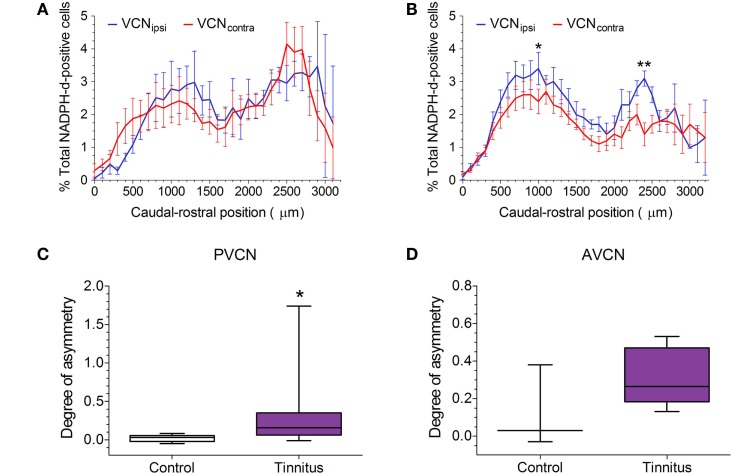
**NOS expression in GPs with suspected tinnitus compared with controls**. **(A)** The mean (±SEM) distribution of NADPH-d-stained cells across the VCN in five un-exposed controls, expressed as a percentage of the total population of counted NADPH-d-positive cells, and plotted according to caudal–rostral position. VCN_ipsi_ is shown in blue and VCN_contra_ in red. **(B)** The mean (±SEM) distribution of NADPH-d-stained cells across the VCN in eight AOE-treated GPs with suspected tinnitus. Data are shown for unilaterally noise-exposed VCN_ipsi_, for which a significantly higher percentage of counted cells was observed, compared with un-exposed VCN_contra_ (*P < 0.05; **P < 0.01). When data were sub-divided according to the bimodal distribution of NADPH-d in VCN, a significant asymmetry was apparent in **(C)** PVCN (*P < 0.05) but not **(D)** AVCN (box and whisker plots show median, interquartile range, and range). Data are expressed as the degree of asymmetry, where values >0 indicate more NADPH-d-positive cells in VCN_ipsi_.

The distribution of NADPH-d-stained neurons across the rostro-caudal axis is shown for pooled control and pooled tinnitus brains in Figures 4A,B, respectively. In both cases, VCN_ipsi_ and VCN_contra_ are shown, expressed as a percentage of the total number of NADPH-d-stained cells counted. As was the case with the single control animal, shown in Figure 2, bimodal distributions were apparent across the extent of the VCN in controls and tinnitus GPs. In control brains, there was a significant effect of rostro-caudal position [two-way ANOVA; *F*(31, 87) = 2.80, *P* < 0.0001], but no significant difference between VCN_ipsi_ and VCN_contra_ [*F*(1, 87) = 0.60, *P* = 0.44] or position × side interaction [*F*(31, 87) = 1.44, *P* = 0.10]. Contrastingly, in tinnitus brains, there was a significant effect of both rostro-caudal position [two-way ANOVA; *F*(32, 174) = 6.17, *P* < 0.0001] and side [*F*(1, 174) = 32.11, *P* < 0.0001], but no position × side interaction [*F*(32, 174) = 1.17, *P* = 0.26]. *Post hoc* analyses revealed significantly more NADPH-d-stained neurons in VCN_ipsi_ at two points across the rostro-caudal axis that suggested changes in both AVCN and PVCN (Figure 4B; *P* < 0.05). Based on these bimodal VCN spatial distribution plots, data were sub-divided into AVCN and PVCN, and statistically assessed separately. The degree of asymmetry between VCN_ipsi_ and VCN_contra_ in tinnitus GPs was significantly greater than controls in PVCN (Figure 4C; Mann–Whitney test; *P* < 0.05). In the AVCN of tinnitus animals, there also appeared to be more NADPH-d-positive cells in VCN_ipsi_ – compared with controls – but variability in control data prevented this difference reaching significance (Figure 4D; Mann–Whitney test; *P* = 0.26). For the VCN as a whole, there was a significant asymmetry as shown previously (22).

### NOS staining intensity and somal size in VCN

**Figure 5:**
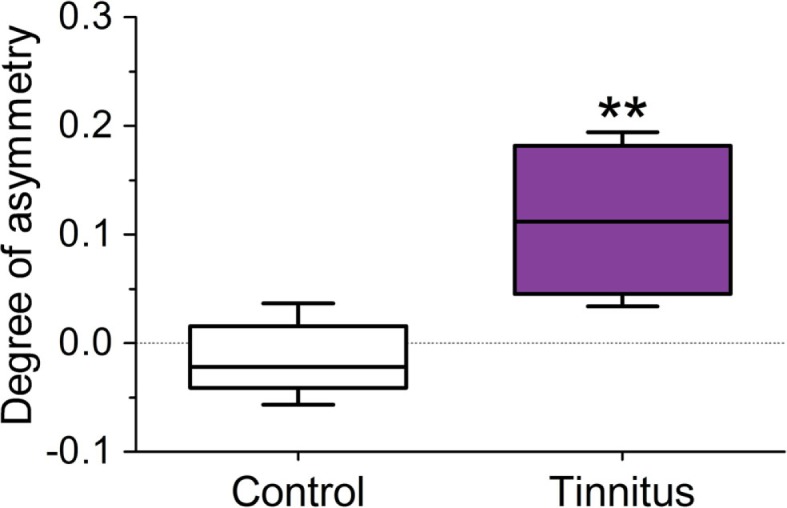
**Changes in the ratio of NADPH-d staining density in the 120 darkest cells between VCN_ipsi_ and VCN_contra_ occur in animals with behavioral evidence of tinnitus**. Tinnitus brains (*n* = 8) were significantly asymmetric in terms of staining density (**P < 0.01) when compared with controls (*n* = 5).

The staining density ratio between VCN_ipsi_ and VCN_contra_ – as measured by examining luminance values across the extent of the VCN – was significantly higher in the tinnitus group (*n* = 8; unpaired *t*-test; *P* < 0.01), when compared with un-exposed controls (Figure 5; *n* = 5). In other words, the amount of NADPH-d in neuronal somata of VCN_ipsi_, relative to VCN_contra_, was substantially higher following AOE and subsequent tinnitus development.

To ascertain whether this asymmetry reflected an increase in NADPH-d staining ipsilaterally, or a decrease contralaterally, optical density in the VCN was expressed relative to a reference standard derived from midline RN for each animal. First, equal numbers of RN were counted in controls on either side of the midline to confirm that there was no asymmetry. There were no statistically significant differences between left- and right-side reticular nuclei, when compared individually within each animal (Mann–Whitney tests; *P* ranged from 0.21 to 0.92). Furthermore, across all control animals (*n* = 5), pooled median luminance values did not exhibit any significant difference between left- and right-side RN (paired *t*-test; *P* = 0.37). Since no left–right differences were detected, RN populations were subsequently pooled for each animal.

Luminance values were converted to a measure of optical density. The darkest cells in control VCN had similar densities to those in the reticular formation; mean (±SEM) density ratio values of 94 ± 3 and 95 ± 3% of RN luminance were observed in VCN_ipsi_ and VCN_contra_, respectively. The mean (±SEM) density ratios across all tinnitus animals (*n* = 8) were 96 ± 5 and 84 ± 4% of reticular density in VCN_ipsi_ and VCN_contra_, respectively.

Normalized VCN_ipsi_ and VCN_contra_ from both control and tinnitus animals were statistically compared with a repeated measures two-way ANOVA. There was no significant effect of experimental group [*F*(1, 11) = 0.52, *P* = 0.49], but significant effects of side [*F*(1, 11) = 9.12, *P* < 0.05], and a group × side interaction [*F*(1, 11) = 14.12, *P* < 0.01] were apparent. A *post hoc* comparison indicated that there were no significant differences between VCN_ipsi_ and VCN_contra_ in controls (*P* = 0.65), but VCN_contra_ was significantly lower than VCN_ipsi_ in tinnitus animals (*P* < 0.01), as would be expected. No further *post hoc* differences were detected when comparing VCN_ipsi_ between control and tinnitus groups (*P* = 0.82), nor VCN_contra_ between control and tinnitus animals (*P* = 0.08). Thus, although VCN percentage ratio values (relative to RN) in tinnitus animals indicated a contralateral reduction was more likely, i.e., 84% in VCN_contra_, this was not statistically significant.

**Figure 6:**
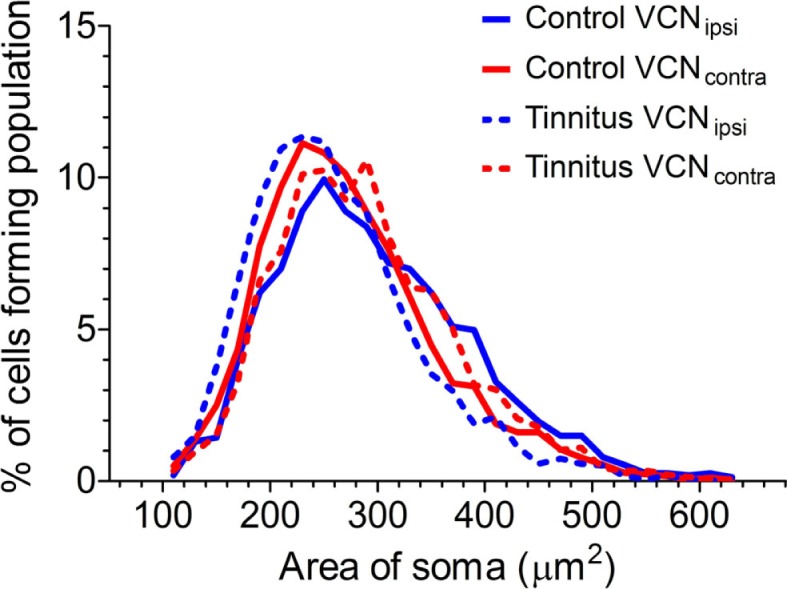
**Comparison of the distributions of cross-sectional area of stained somata in the VCN**. Somal size data from VCN_ipsi_ (shown in blue) and VCN_contra_ (shown in red) are displayed for both control (*n* = 5; solid lines) and tinnitus (*n* = 8; hashed lines) groups of animals, in 20 μm^2^ bins.

Previously, we found evidence of two separate functional populations in the VCN (based on cross-sectional somal area): medium and large multipolar cells. LMs are glycinergic principal neurons, which are bigger than both the excitatory multipolar and bushy neurons (33). In principle, this could have enabled us to detect shifts in the proportions of NOS-containing large inhibitory and smaller excitatory neurons. With this in mind, we measured the area of NADPH-d-stained somata from left and right sides of the brain in all control (Figure 6; *n* = 3338 cells, five GPs) and tinnitus (*n* = 3191 cells, eight GPs) animals. In all cases, i.e., VCN_ipsi_ and VCN_contra_ in both controls and tinnitus GPs, there was a smooth progression of sizes with a single peak ranging between 220 and 280 μm^2^. Since our means of identifying sub-populations relied on detecting discrete groups of cell sizes, it was not possible to identify different populations of NADPH-d-containing neurons based on somal area. In other words, unimodal distribution patterns meant we could not draw conclusions as to the susceptibility of different cell types to AOE, or to subsequent tinnitus development. We also attempted to measure the cross-sectional area of populations of cells that had been identified as either bushy or multipolar cells in control animals, but there were too many cells with ambiguous morphology for us to make any reliable measurement of a complete sub-population.

### Early changes in NOS after AOE

**Figure 7:**
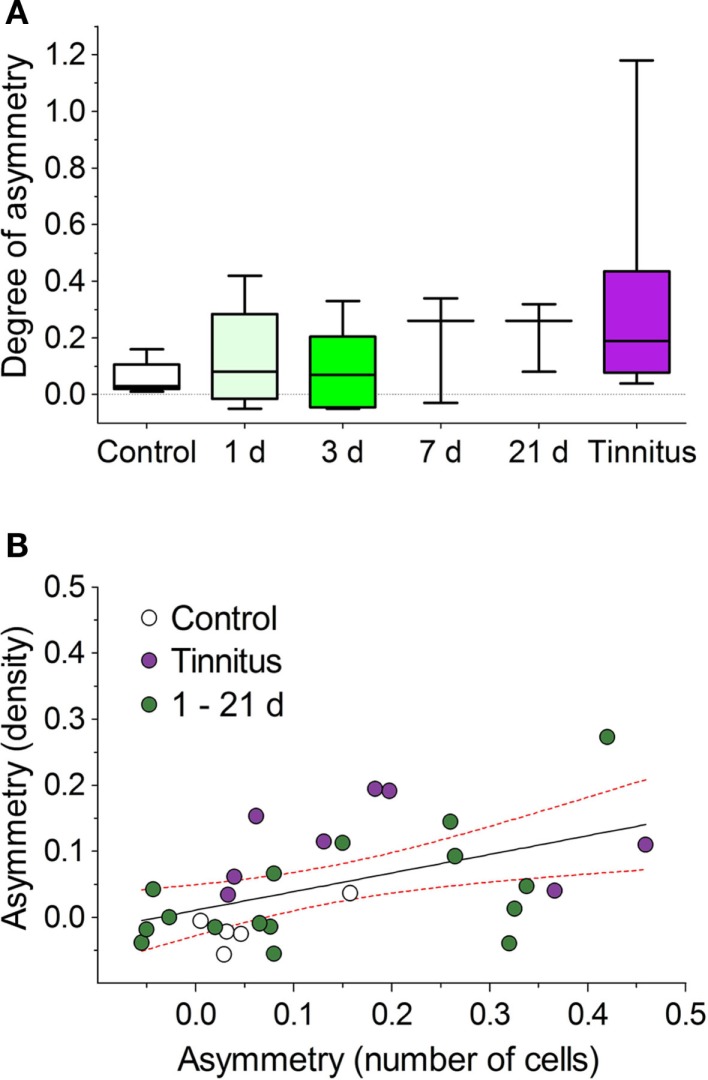
**Variable NOS asymmetries in numbers of stained cells shown for a range of time-points after AOE**. **(A)** Asymmetries between unilaterally noise-exposed VCN_ipsi_ and un-exposed VCN_contra_ are shown for control (*n* = 5), AOE + 1 day (*n* = 5), +3 days (*n* = 5), +7 days (*n* = 3), +21 days (*n* = 3), and tinnitus (*n* = 8) groups. No significant asymmetries were seen when all groups were compared. **(B)** Linear regression analysis comparing relative VCN_ipsi_:VCN_contra_ asymmetries between the number of NADPH-d-positive cells and NADPH-d staining densities from all groups revealed a significant correlation (*r*^2^ = 0.25; *P* < 0.01). Data are shown from all controls (*n* = 5), tinnitus animals (*n* = 8), and combined short-term GPs, i.e., +1, +3, +7, and +21 days groups (*n* = 16). Solid line indicates the results of a linear regression analysis; hashed red lines indicate 95% confidence intervals.

To determine the time course for the onset of AOE-induced NOS changes that might relate to the pathological processes underlying tinnitus, we examined NADPH-d-staining at a series of short-term time-points post-AOE (Figure 7). Large asymmetries between the numbers of stained cells in VCN_ipsi_ and VCN_contra_ were apparent in some individuals (although not in others) as early as 1 day after AOE, and were seen in all other groups (3, 7, and 21 days post-AOE). There were no statistically significant effects across all groups, compared with un-exposed controls (Kruskal–Wallis test; *P* = 0.23), but this was not surprising given the degree of variability between animals (Figure 7A). Despite this, there were large asymmetries in one or two animals at each time-point. These changes were similar in magnitude to that seen in animals with behavioral evidence of tinnitus examined 8 weeks after AOE (Figure 7A). Furthermore, NADPH-d asymmetries were again apparent across the caudal–rostral extent of the VCN in both PVCN and AVCN.

Changes in staining density were also observed in some animals at short-term time-points; a linear regression analysis revealed a significant correlation between asymmetries in cell numbers and asymmetries in staining density (comparing VCN_ipsi_ and VCN_contra_), when short-term time-point animals were included alongside control and tinnitus groups (Figure 7B; *r* = 0.5, *r*^2^ = 0.25; *P* < 0.01). The values for the short-term time-points were scattered around those of the control and tinnitus animals, suggesting that pathways leading to tinnitus development were activated in some animals but not others.

### No correlation between NOS asymmetry and hearing thresholds at short-term time-points

Hearing thresholds were determined with ABR recordings to compare thresholds before AOE, and at the endpoint for each group (data not shown). As expected, shifts were seen in ipsilateral hearing thresholds after AOE in all groups (1, 3, 7, and 21 days), at all frequencies tested (5, 10, and 15 kHz). There were no significant differences in mean threshold shift between AOE groups at either 5 kHz (Kruskal–Wallis test; *P* = 0.16), 10 kHz (Kruskal–Wallis test; *P* = 0.09), or 15 kHz (Kruskal–Wallis test; *P* = 0.16), although the general trend at all frequencies was a predictable improvement in thresholds over time, albeit with considerable variability. Broadband hearing loss, while a little surprising given the narrowband nature of the AOE stimulus, is in keeping with our previous studies using the same noise exposure protocol (22, 37), and other studies demonstrating a mismatch between exposure center frequency and the frequency range of hearing loss (38, 39).

**Figure 8:**
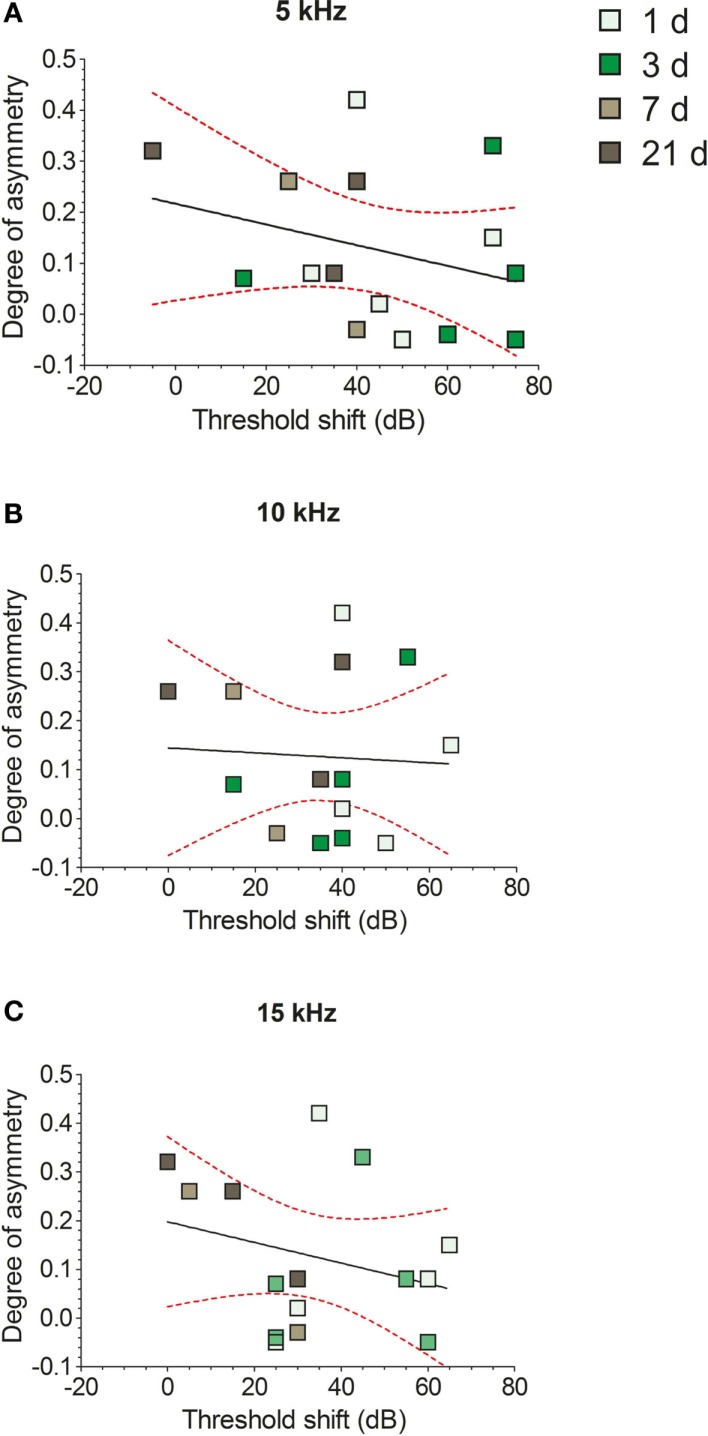
**The effects of unilateral hearing loss on asymmetries in the numbers of NADPH-d-stained cells**. Hearing threshold shifts for each GP – determined by measuring ABRs in response to 5 kHz **(A)**, 10 kHz **(B)**, and 15 kHz **(C)** tones – are plotted against degree of asymmetry for individual GPs. Data from short-term AOE-treated groups of animals are shown (+1, +3, +7, and +21 days). No significant correlations were seen between NADPH-d staining and hearing thresholds.

No significant correlations were found between the degree of NADPH-d staining asymmetry and the extent of hearing loss at either 5 kHz (Figure 8A; *r* = 0.3, *r*^2^ = 0.09, *P* = 0.27), 10 kHz (Figure 8B; *r* = 0.04, *r*^2^ = 0.002, *P* = 0.85), or 15 kHz (Figure 8C; *r* = 0.26, *r*^2^ = 0.07, *P* = 0.33), when examined with a linear regression analysis. This indicates that the relative change in NADPH-d expression in VCN_ipsi_ compared with VCN_contra_ was not proportional to the magnitude of the shift in hearing threshold in each animal.

## Discussion

In the present study, we show that NOS is expressed in a heterogeneous population of morphologically identified principal neurons in the VCN, under normal conditions. Previous work has demonstrated nNOS-positive staining in the CN of rats (23, 40, 41), mice (38), and GPs (22), but the types of neurons involved have not been examined in detail. A proportion of all the main morphological types of neuron in the GP VCN contain NADPH-d, which represents the presence of NOS in aldehyde-fixed tissue (34, 35). Thus, NO-mediated modulation of synaptic strength may potentially be involved in all the main output pathways from the VCN that project to the inferior colliculus, superior olivary complex, and DCN.

Here, we indirectly measured the amount of the enzyme via luminance data, but were only able to make relative comparisons between VCN_ipsi_, VCN_contra_, and the RN within subjects. This was due to variations in tissue fixation, fluctuations in light source intensity, and the degree of background staining. Staining in RN of the medulla served as a standard reference value, constant between animals. By sampling cells at the caudal edge of the DCN, we minimized the possibility of AOE-related effects. This internal reference allowed us to compare differences between animals, however the unpredictable suppression produced by aldehyde fixation meant we could not make any measurement of absolute levels of the enzyme. These data did not reveal any significant, consistent trends with respect to whether NOS increased in VCN_ipsi_, or decreased in VCN_contra_, coincident with the development of tinnitus.

Previous work by Zheng et al. (23) incorporated a quantitative biochemical assay of nNOS present in the rat VCN following salicylate treatment. They found that the number of neurons expressing nNOS increased in the VCN, but overall levels of the enzyme did not appear to change. The reasons for differences between our data and that of Zheng et al. (23) are not entirely clear, although species differences and the likelihood of different mechanisms for salicylate-induced tinnitus and AOE-induced tinnitus are two obvious candidates.

It was unclear in the present study whether NOS changes were predominantly ipsilateral or contralateral. There are a number of pathways by which VCN_ipsi_ could affect the neural processing in the VCN_contra_ (42), and therefore potentially alter NOS expression. The cochlear nucleus (CN) is binaurally sensitive, and about 30% of its neurons are inhibited by contralateral sound stimulation in normal hearing GPs (43, 44). This inhibition is thought to be mediated by large glycinergic multipolar cells, which form a commissural pathway between the VCN on the two sides (45–48). Interestingly, following unilateral conductive hearing loss or cochlear ablation, the number of neurons in the VCN that produce an excitatory response when stimulated by the contralateral ear increases by a factor of 10 (49). This increase in the level of excitation occurs within a few hours and is thought to be due to an upregulation of existing excitatory pathways. This could involve both direct commissural glutamatergic fibers and an indirect pathway involving cholinergic neurons sending collaterals from the olivocochlear bundle (42). Neuroplastic changes such as these would probably involve alterations in second messenger systems contralaterally, which may explain alterations in VCN_contra_ NOS levels, as well as in VCN_ipsi_. The cholinergic input to VCN arises from multiple groups of cholinergic neurons in the tegmentum that include the laterodorsal tegmental nucleus, pedunculopontine nucleus, and superior olivary complex, all of which are thought to have a role in sensory gating and possibly tinnitus (50). These cholinergic inputs appear to be selective and mainly target the medium multipolar cells (MM) that project via the trapezoid body while not influencing other cell types (51).

Principal neurons in the VCN are largely glutamatergic; this includes SC and GC, small-to-medium-sized multipolar or stellate cells, and octopus cells (30, 52). Conversely, the largest cells in the VCN are glycinergic multipolar neurons that exhibit “onset”-type firing patterns in response to auditory stimuli (33, 47). Unfortunately, we were not able to determine which types of neurons were involved in AOE-related changes in the present study, based on soma area measurements. There appeared to be subtle differences in cell size distributions, which might reflect shifts in the types of cell expressing NOS, but we cannot say with these data. Future studies should utilize co-labeling with nNOS and either glutamatergic or glycinergic markers to elucidate the localization of AOE-related changes in NOS. Furthermore, physiological studies would address the functional nature of fluctuating NOS, in terms of modifying the balance between excitation and inhibition.

There have been very few studies of NO function in the VCN but it is reasonable to assume that it has similar roles to those found in other parts of the brain. NO, produced post-synaptically by nNOS, acts as a retrograde neuromodulator at pre-synaptic sites to regulate plasticity in the brain and can contribute to either long-term potentiation or long-term depression, depending on local neural circuitry (53–57). A number of studies indicate that NO can also act post-synaptically to modulate long-term potentiation (58, 59). NO generation by nNOS is calcium-dependent, and is commonly associated with calcium influx via NMDA receptor-mediated ion channels. However, a study of the mouse VCN demonstrated high levels of a splice variant of nNOS that is not associated with NMDA receptors and may be associated with a different activation mechanism (60). In the medial nucleus of the trapezoid body of mice, NO appeared to modulate excitability in a number of adjacent neurons, in an activity-dependent fashion. This effect was exerted predominantly via manipulation of specific potassium channels, altering action potential characteristics and reducing the fidelity of synaptic transmission, and was proposed to be a gain control mechanism utilized during periods of intense neuronal input (61).

Further studies are needed to establish how NO production in the VCN relates to the pathophysiological changes associated with AOE, or the subsequent development of tinnitus. A framework for these studies might be provided by considering the role NO is thought to have in the development of chronic, neuropathic pain (62–64), which has been specifically linked to nNOS (65). Neuropathic pain shares some common attributes with tinnitus: both are phantom sensory percepts believed to originate peripherally through deafferentation, and to subsequently involve central mechanisms [see Ref. (66) for a review].

In light of findings from models of neuropathic pain, and a considerable body of work identifying a key role for NO in regulating plasticity in other brain areas, it seems plausible that changes in NO-mediated neuromodulation could have altered neuronal activity in the VCN of our AOE-exposed GPs. There is already evidence demonstrating changes in inhibitory GABAergic and glycinergic signaling in models of tinnitus, with specific effects in the VCN (7, 8, 67–69). Moreover, morphological changes – largely specific to excitatory synapses – in the VCN have been identified following AOE (67, 70–72). Accordingly, it seems likely that the VCN plays a role in the pathogenesis of tinnitus.

A prevalent model of tinnitus generation proposes a change in the gain control mechanisms of the brainstem: decreased auditory afferent input, occurring as a result of peripheral damage, is compensated for by increasing the gain mediated by synaptic connections in the brainstem (1, 2). The principle confound to its validity lies in explaining how only a proportion of individuals experience tinnitus, when taken from a group of people all displaying similar degrees and types of peripheral damage and subsequent hearing loss (15, 16). The same is true in animal studies, using a variety of small-animal models; although the relative proportions of “tinnitus” and “no-tinnitus” animals varies, most report a number of individuals that do not develop tinnitus following induction by AOE (73, 74). Thus, to be plausible, the model should allow the same degree of peripheral hearing loss to produce different effects across a population.

Nitric oxide production represents a potential candidate for the variable that determines whether or not tinnitus develops. Enhanced NO signaling or nNOS expression at least, was associated with positive behavioral evidence of tinnitus in salicylate-treated rats (23), while altered NOS expression was associated with behavioral tinnitus in AOE-treated GPs (22). GPs without behavioral evidence of tinnitus did not exhibit any evidence of altered NO signaling. NO could conceivably regulate the balance of excitation and inhibition through being elevated in excitatory neurons, yet diminished in inhibitory neurons, thus contributing to gain changes that trigger tinnitus-related hyperactivity. Here, we showed that changes in NOS were apparent as early as 24 h after AOE, implying that NO-related plastic changes occur within a short time-frame after acoustic insult. Interestingly, there was a notable variability; only a proportion of animals exhibited large ipsilateral–contralateral asymmetries at each time-point. This variability was not linked to the degree of hearing loss which was independently variable. The variability in NOS expression, coupled with the dichotomy between tinnitus and no-tinnitus animals in our previous study, supports the possibility that the mechanisms underlying the expression of NOS could account for inter-subject differences when considering the link between AOE, peripheral damage, and tinnitus development.

In the present study, we focused on the VCN. Clearly – since the VCN does not function in isolation – pathological changes that produce a tinnitus percept likely involve abnormal propagation of activity through multiple levels of the brain (75). The DCN is also thought to be involved in central hyperactivity that might underlie elevated central gain (6, 76–78). The DCN and VCN are linked; large glycinergic multipolar neurons provide local inhibition in the VCN, but are thought to also provide widespread inhibition in the DCN (79). Consequently, altered signaling in large multipolar neurons could also decrease the level of inhibition in the DCN.

To conclude, NO is a potential contributing factor to the central gain model of generation and maintenance of tinnitus. Variability between individuals at short-term time-points, and the dichotomy between tinnitus and no-tinnitus animals, reported in our previous study (22), lend support to the idea that the neuromodulation system involving NO could determine the progressive changes that follow AOE, which ultimately either do or do not result in tinnitus.

## Author Contributions

BC, JB, AP, and MW designed the study; BC, JB, and MW acquired the data; BC, VK, JB, and MW analyzed and interpreted the data; and BC, AP, and MW wrote and edited the manuscript.

## Conflict of Interest Statement

The authors declare that the research was conducted in the absence of any commercial or financial relationships that could be construed as a potential conflict of interest.

## Abbreviations

ABR, auditory brainstem response; AOE, acoustic over-exposure; AVCN, anterior ventral cochlear nucleus; CN, cochlear nucleus; DAB, diaminobenzidine; DCN, dorsal cochlear nucleus; GC, globular bushy cell; GP, guinea pig; LM, large multipolar cell; MM, medium multipolar cell; NADPH-d, reduced nicotinamide adenine dinucleotide phosphate diaphorase; NHS, normal horse serum; nNOS, neuronal nitric oxide synthase; NO, nitric oxide; NOS, nitric oxide synthase; OC, octopus cell; PB, phosphate buffer; PVCN, posterior ventral cochlear nucleus; RN, reticular neurons; SC, spherical bushy cell; TDT, Tucker Davis Technologies; VCN, ventral cochlear nucleus.
